# (*S*_P_,*S*_P_)-(–)-(*E*)-1,2-Bis(methyl­phenyl­phosphino­yl)ethene

**DOI:** 10.1107/S1600536809004632

**Published:** 2009-02-13

**Authors:** Holger Butenschön, Nikolai Vinokurov, Ingmar Baumgardt, K. Michal Pietrusiewicz

**Affiliations:** a Institut für Organische Chemie, Leibniz Universität Hannover, Schneiderberg 1B, 30167 Hannover, Germany; b Department of Organic Chemistry, Maria Curie-Sklodowska-University Lublin, ul. Gliniana 33, Lublin, PL-20-614, Poland

## Abstract

The title compound, C_16_H_18_O_2_P_2_, possesses two stereogenic P atoms and shows a distorted *s*–*cis* conformation of each O=P—C=C moiety. This has been suggested on the basis of the stereochemical result of 1,3-dipolar cyclo­additions with nitro­nes and is now confirmed by the present crystal structure analysis. There are two crystallographically independent molecules in the asymmetric unit.

## Related literature

For optically active *P*-stereogenic 1,2-diphosphinoethanes and diphosphane dioxides, see: Crepy & Imamoto (2003*a* ▶,*b* ▶); Glueck (2008 ▶); Knowles (1983 ▶, 2002 ▶); Pietrusiewicz & Zablocka (1988 ▶); Demchuk *et al.* (2003 ▶); Vinokurov *et al.* (2006 ▶); Vinokurov, Garabatos-Perera *et al.* (2008 ▶) and Vinokurov, Pietrusiewicz *et al.* (2008 ▶). For the structures of (–)-(*S*_P_)-methyl­phenyl­phosphine oxide and (+)-(*R*_P_)-(*tert*-butyl­vinyl­phosphino­yl)benzene, see: Pietrusiewicz *et al.* (1991 ▶); Szmigielska *et al.* (2006 ▶). For the determination of the absolute configuration of the stereogenic centers for the title compound, see: Pietrusiewicz *et al.* (1984 ▶, 1991 ▶) and Vinokurov, Pietrusiewicz *et al.* (2008 ▶).
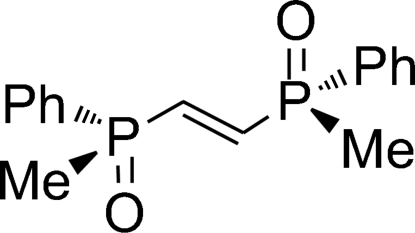


## Experimental

### 

#### Crystal data

C_16_H_18_O_2_P_2_
*M* *_r_* = 304.24
Monoclinic, 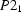

*a* = 11.686 (5) Å
*b* = 5.5291 (15) Å
*c* = 24.132 (10) Å
β = 96.36 (5)°
*V* = 1549.7 (10) Å^3^
*Z* = 4
Mo *K*α radiation
μ = 0.28 mm^−1^
*T* = 297 K
0.35 × 0.29 × 0.18 mm

#### Data collection

Stoe IPDS diffractometer
Absorption correction: multi-scan (Blessing, 1995 ▶) *T*_min_ = 0.927, *T*_max_ = 0.953
20851 measured reflections
6041 independent reflections
4640 reflections with *I* > 2σ(*I*)
*R*_int_ = 0.067

#### Refinement

*R*[*F* ^2^ > 2σ(*F* ^2^)] = 0.041
*wR*(*F* ^2^) = 0.097
*S* = 0.99
6041 reflections
361 parameters
1 restraint
H-atom parameters constrained
Δρ_max_ = 0.37 e Å^−3^
Δρ_min_ = −0.19 e Å^−3^
Absolute structure: Flack (1983 ▶), 2656 Friedel pairs
Flack parameter: 0.01 (9)

### 

Data collection: *IPDS* (Stoe & Cie, 1999 ▶); cell refinement: *IPDS*; data reduction: *IPDS*; program(s) used to solve structure: *SHELXS97* (Sheldrick, 2008 ▶) and *WinGX* (Farrugia, 1999 ▶); program(s) used to refine structure: *SHELXL97* (Sheldrick, 2008 ▶); molecular graphics: *ORTEP-3* (Farrugia, 1997 ▶); software used to prepare material for publication: *enCIFer* (Allen *et al.*, 2004 ▶) and *publCIF* (Westrip, 2009 ▶).

## Supplementary Material

Crystal structure: contains datablocks I, global. DOI: 10.1107/S1600536809004632/jh2072sup1.cif

Structure factors: contains datablocks I. DOI: 10.1107/S1600536809004632/jh2072Isup2.hkl

Additional supplementary materials:  crystallographic information; 3D view; checkCIF report

## Figures and Tables

**Fig. 1.**
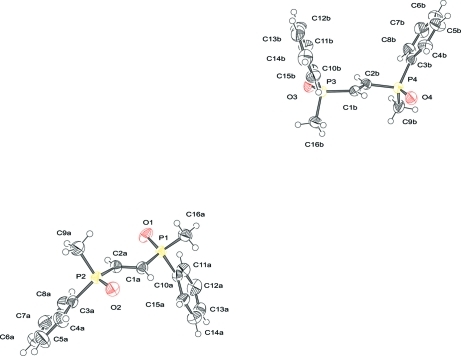
Structure of (1) in the crystal with atom labels and 50% probability displacement ellipsoids for non-H atoms.

## supplementary crystallographic information

### Comment

Optically active P-stereogenic 1,2-diphosphinoethanes constitute an important class of chiral bidentate ligands of great practical utility in the field of asymmetric catalysis (Crepy & Imamoto, 2003, Glueck, 2008, Knowles, 1983, 2002). The corresponding P-stereogenic diphosphane dioxides, which are the most direct precursors to such ligands, have recently been shown to be easily accessible through a simple conjugate addition of secondary phosphine oxides to the homochiral (–)-(*S*_P_)-methylphenylphosphine oxide oxide (Pietrusiewicz & Zablocka, 1988). Recently, we reported on the synthesis of (*S*_P_,*S*_P_)-(–)-(*E*)-ethene-1,2-diylbis[methyl(phenyl)phosphine] dioxide (1) by the homo cross-metathesis reaction of (*S*)-methylphenylvinylphosphine oxide (Demchuk *et al.*, 2003, Vinokurov *et al.*, 2008, Vinokurov *et al.*, 2006) and then studied the reactivity of 1 in 1,3-dipolar cycloadditions with acyclic nitrones to achieve new bidentate P-stereogenic phosphane ligands after stereospecific reduction (Vinokurov *et al.*, 2008).

Although we have recently postulated the di-*s*-cis conformation of 1 as the reactive conformation in the thermal 1,3-dipolar cycloaddition, no experimental evidence with regard to the conformation of 1 has yet been reported. However, the structure of the related (–)-(*S*_P_)-methylphenylphosphine oxide (Pietrusiewicz *et al.*, 1991) and (+)-(*R*_P_)-(*tert*-butylvinylphosphinoyl)benzene have recently been reported (Szmigielska *et al.*, 2006). Herein, we describe the solid state structure of (*S*_P_,*S*_P_)-(–)-(*E*)-ethene-1,2-diylbis[methyl(phenyl)phosphine] dioxide (1), which has been obtained by a single-crystal X-ray structure analysis.

The molecular structure of 1 is displayed in Fig. 1. The absolute configuration of the stereogenic centers has not been determined crystallographically but is evident from that of the starting material (Pietrusiewicz *et al.*, 1984, Pietrusiewicz *et al.*, 1991) as well as from the crystal structure analysis of a cycloaddition product (Vinokurov *et al.*, 2008). The largest substituents of each phosphorus atom are placed in the most distant zigzag positions, and the P1=O1 and P2=O2 dipoles are oriented in opposite directions relative to one another. The deviation from planarity of the O=P—C=C—P=O is reflected by the torsional angles given in Table 1.

As typical for compounds of type R_3_P=O deformations of the tetrahedral environment of the P atoms cause an increase of the O—P—C and the simultaneous decrease of the C—P—C valency angles. The values observed fall in the range of 110.28 (15) - 115.80 (17)° and 101.69 (17) - 107.26 (15)° for P1 and, 110.87 (15)° - 114.73 (15)° and 102.42 (17) - 107.94 (14)° for P2, respectively.

DFT calculations show the observed conformation to be more stable than the corresponding di-*s*-trans conformation by 16.54 kJ/mol (TURBOMOLE 5.7 Method BP86/SV(P).

### Experimental

For the preparation of (*S*_P_,*S*_P_)-(-)-(*E*)-1,2-bis(methylphenylphosphinoyl)ethene (1) see Vinokurov *et al.* (2006).

### Refinement

Note: The asymmetric unit contains two crystallographically independent molecules, one of which is presented here. H atoms were positioned geometrically and refined using a riding model with C—H = 0.93–0.96 Å and with *U*_iso_(H) = 1.2 (1.5 for methyl groups) × *U*_eq_(C).

### Crystal data

**Table:**

C_16_H_18_O_2_P_2_	*F*(000) = 640
*M**_r_* = 304.24	*D*_x_ = 1.304 Mg m^−^^3^
Monoclinic, *P*2_1_	Melting point: 511 K
Hall symbol: P 2yb	Mo *K*α radiation, λ = 0.71073 Å
*a* = 11.686 (5) Å	Cell parameters from 8000 reflections
*b* = 5.5291 (15) Å	θ = 2.3–25.4°
*c* = 24.132 (10) Å	µ = 0.28 mm^−^^1^
β = 96.36 (5)°	*T* = 297 K
*V* = 1549.7 (10) Å^3^	Plate, white
*Z* = 4	0.35 × 0.29 × 0.18 mm

### Data collection

**Table:**

Stoe IPDS diffractometer	6041 independent reflections
Radiation source: fine-focus sealed tube	4640 reflections with *I* > 2σ(*I*)
graphite	*R*_int_ = 0.067
psi scans	θ_max_ = 26.0°, θ_min_ = 2.3°
Absorption correction: multi-scan (Blessing, 1995)	*h* = −14→14
*T*_min_ = 0.927, *T*_max_ = 0.953	*k* = −6→6
20851 measured reflections	*l* = −29→29

### Refinement

**Table:**

Refinement on *F*^2^	Secondary atom site location: difference Fourier map
Least-squares matrix: full	Hydrogen site location: inferred from neighbouring sites
*R*[*F*^2^ > 2σ(*F*^2^)] = 0.041	H-atom parameters constrained
*wR*(*F*^2^) = 0.097	*w* = 1/[σ^2^(*F*_o_^2^) + (0.0482*P*)^2^] where *P* = (*F*_o_^2^ + 2*F*_c_^2^)/3
*S* = 0.99	(Δ/σ)_max_ = 0.002
6041 reflections	Δρ_max_ = 0.37 e Å^−^^3^
361 parameters	Δρ_min_ = −0.19 e Å^−^^3^
1 restraint	Absolute structure: Flack (1983), 2656 Friedel pairs
Primary atom site location: structure-invariant direct methods	Flack parameter: 0.01 (9)

### Special details

**Table:**

Geometry. All e.s.d.'s (except the e.s.d. in the dihedral angle between two l.s. planes) are estimated using the full covariance matrix. The cell e.s.d.'s are taken into account individually in the estimation of e.s.d.'s in distances, angles and torsion angles; correlations between e.s.d.'s in cell parameters are only used when they are defined by crystal symmetry. An approximate (isotropic) treatment of cell e.s.d.'s is used for estimating e.s.d.'s involving l.s. planes.
Refinement. Refinement of *F*^2^ against ALL reflections. The weighted *R*-factor *wR* and goodness of fit *S* are based on *F*^2^, conventional *R*-factors *R* are based on *F*, with *F* set to zero for negative *F*^2^. The threshold expression of *F*^2^ > σ(*F*^2^) is used only for calculating *R*-factors(gt) *etc*. and is not relevant to the choice of reflections for refinement. *R*-factors based on *F*^2^ are statistically about twice as large as those based on *F*, and *R*- factors based on ALL data will be even larger.

### Fractional atomic coordinates and isotropic or equivalent isotropic displacement parameters (Å^2^)

**Table:**

	*x*	*y*	*z*	*U*_iso_*/*U*_eq_	
P1	0.30457 (7)	0.21685 (14)	0.97574 (3)	0.0353 (2)	
P2	0.39031 (6)	−0.02618 (15)	1.14954 (3)	0.0346 (2)	
O1	0.3363 (2)	0.4760 (5)	0.98268 (10)	0.0538 (6)	
O2	0.3896 (2)	−0.2919 (4)	1.14004 (10)	0.0470 (6)	
C1A	0.3199 (3)	0.0452 (6)	1.03959 (12)	0.0390 (7)	
H1A	0.3089	−0.1213	1.0382	0.047*	
C2A	0.3466 (3)	0.1518 (6)	1.08850 (12)	0.0377 (7)	
H2A	0.3427	0.3193	1.0913	0.045*	
C3A	0.2989 (3)	0.0536 (6)	1.20236 (11)	0.0379 (7)	
C4A	0.2921 (4)	−0.1114 (7)	1.24450 (15)	0.0600 (10)	
H4A	0.3340	−0.2545	1.2446	0.072*	
C5A	0.2234 (5)	−0.0671 (11)	1.28698 (17)	0.0792 (14)	
H5A	0.2188	−0.1808	1.3150	0.095*	
C6A	0.1639 (4)	0.1403 (10)	1.28732 (19)	0.0741 (13)	
H6A	0.1185	0.1701	1.3159	0.089*	
C7A	0.1693 (4)	0.3089 (9)	1.2460 (2)	0.0704 (12)	
H7A	0.1271	0.4512	1.2464	0.085*	
C8A	0.2380 (3)	0.2673 (8)	1.20324 (15)	0.0531 (8)	
H8A	0.2427	0.3827	1.1755	0.064*	
C9A	0.5286 (3)	0.0957 (8)	1.17267 (15)	0.0557 (10)	
H9A1	0.5606	0.0118	1.2056	0.083*	
H9A2	0.5212	0.2645	1.1809	0.083*	
H9A3	0.5784	0.0765	1.1439	0.083*	
C10A	0.1564 (3)	0.1880 (6)	0.94567 (12)	0.0355 (7)	
C11A	0.1106 (3)	0.3729 (7)	0.91121 (15)	0.0525 (9)	
H11A	0.1567	0.5032	0.9036	0.063*	
C12A	−0.0027 (4)	0.3649 (8)	0.88820 (19)	0.0639 (11)	
H12A	−0.0319	0.4875	0.8643	0.077*	
C13A	−0.0721 (3)	0.1788 (9)	0.90020 (18)	0.0625 (11)	
H13A	−0.1492	0.1782	0.8856	0.075*	
C14A	−0.0288 (3)	−0.0100 (9)	0.93402 (17)	0.0644 (11)	
H14A	−0.0760	−0.1382	0.9417	0.077*	
C15A	0.0867 (3)	−0.0058 (7)	0.95651 (13)	0.0490 (8)	
H15A	0.1169	−0.1329	0.9788	0.059*	
C16A	0.3893 (3)	0.0436 (8)	0.93298 (14)	0.0508 (9)	
H16A	0.3879	0.1190	0.8971	0.076*	
H16B	0.3583	−0.1170	0.9286	0.076*	
H16C	0.4672	0.0355	0.9503	0.076*	
P3	0.69714 (6)	0.45422 (16)	0.52266 (3)	0.03485 (19)	
P4	0.61341 (7)	0.25487 (16)	0.34535 (3)	0.0377 (2)	
O3	0.6815 (2)	0.7189 (5)	0.51686 (10)	0.0521 (6)	
O4	0.6118 (2)	−0.0103 (5)	0.35230 (10)	0.0528 (6)	
C1B	0.6767 (2)	0.2977 (6)	0.45711 (12)	0.0363 (7)	
H1B	0.6813	0.1298	0.4570	0.044*	
C2B	0.6553 (2)	0.4128 (6)	0.40913 (11)	0.0361 (7)	
H2B	0.6621	0.5803	0.4085	0.043*	
C3B	0.7090 (3)	0.3418 (6)	0.29501 (12)	0.0395 (7)	
C4B	0.7920 (3)	0.1795 (7)	0.28267 (15)	0.0518 (9)	
H4B	0.8021	0.0365	0.3029	0.062*	
C5B	0.8607 (3)	0.2254 (9)	0.24069 (18)	0.0677 (11)	
H5B	0.9151	0.1122	0.2324	0.081*	
C6B	0.8484 (4)	0.4391 (9)	0.21121 (17)	0.0662 (11)	
H6B	0.8953	0.4718	0.1834	0.079*	
C7B	0.7671 (4)	0.6022 (8)	0.22304 (18)	0.0679 (12)	
H7B	0.7576	0.7451	0.2027	0.082*	
C8B	0.6986 (4)	0.5562 (7)	0.26523 (16)	0.0584 (10)	
H8B	0.6449	0.6709	0.2736	0.070*	
C9B	0.4772 (3)	0.3870 (7)	0.32300 (15)	0.0547 (10)	
H9B1	0.4460	0.3149	0.2884	0.082*	
H9B2	0.4864	0.5578	0.3179	0.082*	
H9B3	0.4256	0.3595	0.3507	0.082*	
C10B	0.8407 (3)	0.3773 (6)	0.55361 (12)	0.0345 (7)	
C11B	0.9281 (3)	0.5428 (8)	0.54766 (15)	0.0542 (9)	
H11B	0.9113	0.6854	0.5280	0.065*	
C12B	1.0393 (3)	0.4980 (9)	0.57053 (19)	0.0678 (12)	
H12B	1.0971	0.6092	0.5658	0.081*	
C13B	1.0648 (3)	0.2913 (9)	0.60005 (18)	0.0650 (12)	
H13B	1.1397	0.2632	0.6160	0.078*	
C14B	0.9795 (3)	0.1226 (9)	0.60642 (17)	0.0634 (11)	
H14B	0.9971	−0.0187	0.6265	0.076*	
C15B	0.8674 (3)	0.1661 (7)	0.58256 (16)	0.0508 (9)	
H15B	0.8103	0.0520	0.5862	0.061*	
C16B	0.5972 (3)	0.3059 (8)	0.56197 (14)	0.0520 (9)	
H16D	0.6010	0.3767	0.5984	0.078*	
H16E	0.6161	0.1371	0.5653	0.078*	
H16F	0.5207	0.3239	0.5433	0.078*	

### Atomic displacement parameters (Å^2^)

**Table:**

	*U*^11^	*U*^22^	*U*^33^	*U*^12^	*U*^13^	*U*^23^
P1	0.0399 (4)	0.0320 (5)	0.0331 (4)	−0.0040 (3)	0.0008 (3)	0.0008 (3)
P2	0.0375 (4)	0.0341 (5)	0.0315 (4)	−0.0016 (4)	0.0000 (3)	0.0002 (3)
O1	0.0653 (15)	0.0310 (14)	0.0615 (14)	−0.0135 (12)	−0.0088 (12)	0.0025 (12)
O2	0.0612 (14)	0.0282 (13)	0.0521 (12)	0.0006 (11)	0.0089 (11)	−0.0009 (10)
C1A	0.0425 (17)	0.0376 (17)	0.0362 (15)	−0.0016 (14)	0.0015 (13)	0.0010 (13)
C2A	0.0434 (17)	0.0346 (17)	0.0349 (15)	0.0013 (13)	0.0036 (13)	0.0025 (12)
C3A	0.0377 (16)	0.0416 (17)	0.0331 (14)	−0.0029 (14)	−0.0024 (12)	−0.0020 (13)
C4A	0.080 (3)	0.054 (3)	0.0479 (19)	0.0009 (19)	0.0144 (19)	0.0109 (16)
C5A	0.112 (4)	0.083 (4)	0.048 (2)	−0.004 (3)	0.032 (2)	0.006 (2)
C6A	0.074 (3)	0.086 (3)	0.068 (3)	−0.015 (3)	0.034 (2)	−0.016 (2)
C7A	0.056 (2)	0.067 (3)	0.092 (3)	0.002 (2)	0.024 (2)	−0.014 (2)
C8A	0.052 (2)	0.046 (2)	0.062 (2)	0.0021 (17)	0.0120 (16)	0.0027 (17)
C9A	0.045 (2)	0.070 (3)	0.0511 (19)	−0.0026 (18)	−0.0025 (16)	0.0010 (19)
C10A	0.0431 (16)	0.0322 (18)	0.0312 (13)	0.0015 (13)	0.0032 (12)	−0.0009 (12)
C11A	0.054 (2)	0.046 (2)	0.057 (2)	0.0032 (16)	−0.0003 (17)	0.0054 (16)
C12A	0.058 (2)	0.059 (3)	0.070 (3)	0.014 (2)	−0.014 (2)	0.002 (2)
C13A	0.043 (2)	0.070 (3)	0.071 (2)	0.0076 (19)	−0.0055 (18)	−0.024 (2)
C14A	0.051 (2)	0.077 (3)	0.066 (2)	−0.015 (2)	0.0072 (18)	−0.007 (2)
C15A	0.0511 (18)	0.045 (2)	0.0483 (17)	−0.0064 (16)	−0.0042 (14)	0.0050 (16)
C16A	0.0446 (19)	0.059 (2)	0.0503 (18)	−0.0003 (16)	0.0116 (15)	0.0019 (17)
P3	0.0352 (4)	0.0359 (5)	0.0330 (4)	0.0013 (4)	0.0018 (3)	−0.0026 (4)
P4	0.0410 (4)	0.0376 (5)	0.0345 (4)	−0.0042 (4)	0.0039 (3)	−0.0058 (4)
O3	0.0566 (15)	0.0390 (15)	0.0578 (14)	0.0047 (12)	−0.0064 (11)	−0.0032 (12)
O4	0.0715 (16)	0.0363 (15)	0.0538 (13)	−0.0085 (12)	0.0205 (12)	−0.0051 (11)
C1B	0.0320 (15)	0.0399 (19)	0.0369 (15)	−0.0027 (13)	0.0025 (12)	−0.0022 (13)
C2B	0.0339 (15)	0.0399 (19)	0.0346 (14)	0.0015 (13)	0.0040 (12)	−0.0015 (13)
C3B	0.0430 (17)	0.0439 (18)	0.0303 (14)	−0.0042 (14)	−0.0020 (13)	−0.0005 (13)
C4B	0.0444 (18)	0.052 (2)	0.059 (2)	0.0056 (16)	0.0084 (16)	0.0110 (17)
C5B	0.053 (2)	0.077 (3)	0.077 (3)	0.008 (2)	0.024 (2)	0.000 (3)
C6B	0.072 (3)	0.072 (3)	0.059 (2)	−0.017 (3)	0.027 (2)	0.002 (2)
C7B	0.095 (3)	0.054 (3)	0.058 (2)	−0.002 (2)	0.022 (2)	0.012 (2)
C8B	0.079 (3)	0.042 (2)	0.058 (2)	0.0048 (19)	0.0219 (19)	0.0017 (18)
C9B	0.0432 (18)	0.065 (3)	0.0533 (19)	0.0008 (17)	−0.0058 (15)	−0.0185 (18)
C10B	0.0341 (15)	0.0381 (18)	0.0310 (14)	0.0000 (12)	0.0027 (12)	−0.0041 (12)
C11B	0.0449 (19)	0.056 (2)	0.062 (2)	−0.0049 (16)	0.0059 (16)	0.0030 (18)
C12B	0.0373 (19)	0.078 (3)	0.087 (3)	−0.013 (2)	0.0034 (18)	−0.011 (3)
C13B	0.041 (2)	0.079 (3)	0.071 (2)	0.015 (2)	−0.0086 (17)	−0.025 (2)
C14B	0.057 (2)	0.067 (3)	0.062 (2)	0.018 (2)	−0.0108 (18)	−0.002 (2)
C15B	0.0440 (19)	0.047 (2)	0.060 (2)	0.0004 (15)	0.0014 (16)	0.0042 (17)
C16B	0.0427 (18)	0.067 (3)	0.0485 (19)	−0.0010 (17)	0.0128 (15)	0.0009 (17)

### Geometric parameters (Å, °)

**Table:**

P1—O1	1.485 (3)	P3—O3	1.480 (3)
P1—C16A	1.786 (4)	P3—C16B	1.784 (3)
P1—C1A	1.801 (3)	P3—C1B	1.796 (3)
P1—C10A	1.809 (3)	P3—C10B	1.810 (3)
P2—O2	1.487 (3)	P4—O4	1.476 (3)
P2—C9A	1.782 (4)	P4—C9B	1.780 (4)
P2—C2A	1.798 (3)	P4—C2B	1.790 (3)
P2—C3A	1.806 (3)	P4—C3B	1.804 (3)
C1A—C2A	1.325 (4)	C1B—C2B	1.321 (4)
C1A—H1A	0.9300	C1B—H1B	0.9300
C2A—H2A	0.9300	C2B—H2B	0.9300
C3A—C4A	1.375 (5)	C3B—C4B	1.378 (5)
C3A—C8A	1.381 (5)	C3B—C8B	1.385 (5)
C4A—C5A	1.392 (6)	C4B—C5B	1.384 (5)
C4A—H4A	0.9300	C4B—H4B	0.9300
C5A—C6A	1.341 (7)	C5B—C6B	1.378 (7)
C5A—H5A	0.9300	C5B—H5B	0.9300
C6A—C7A	1.372 (7)	C6B—C7B	1.363 (6)
C6A—H6A	0.9300	C6B—H6B	0.9300
C7A—C8A	1.394 (5)	C7B—C8B	1.387 (5)
C7A—H7A	0.9300	C7B—H7B	0.9300
C8A—H8A	0.9300	C8B—H8B	0.9300
C9A—H9A1	0.9600	C9B—H9B1	0.9600
C9A—H9A2	0.9600	C9B—H9B2	0.9600
C9A—H9A3	0.9600	C9B—H9B3	0.9600
C10A—C11A	1.387 (5)	C10B—C15B	1.379 (5)
C10A—C15A	1.388 (5)	C10B—C11B	1.391 (5)
C11A—C12A	1.378 (6)	C11B—C12B	1.377 (5)
C11A—H11A	0.9300	C11B—H11B	0.9300
C12A—C13A	1.361 (6)	C12B—C13B	1.362 (7)
C12A—H12A	0.9300	C12B—H12B	0.9300
C13A—C14A	1.386 (6)	C13B—C14B	1.386 (6)
C13A—H13A	0.9300	C13B—H13B	0.9300
C14A—C15A	1.397 (5)	C14B—C15B	1.392 (5)
C14A—H14A	0.9300	C14B—H14B	0.9300
C15A—H15A	0.9300	C15B—H15B	0.9300
C16A—H16A	0.9600	C16B—H16D	0.9600
C16A—H16B	0.9600	C16B—H16E	0.9600
C16A—H16C	0.9600	C16B—H16F	0.9600
			
O1—P1—C16A	115.80 (17)	O3—P3—C16B	115.05 (17)
O1—P1—C1A	114.32 (15)	O3—P3—C1B	112.97 (15)
C16A—P1—C1A	101.69 (17)	C16B—P3—C1B	102.46 (17)
O1—P1—C10A	110.28 (15)	O3—P3—C10B	111.77 (15)
C16A—P1—C10A	106.75 (16)	C16B—P3—C10B	107.71 (16)
C1A—P1—C10A	107.26 (15)	C1B—P3—C10B	106.10 (14)
O2—P2—C9A	114.30 (18)	O4—P4—C9B	114.84 (18)
O2—P2—C2A	114.73 (15)	O4—P4—C2B	113.11 (15)
C9A—P2—C2A	102.42 (17)	C9B—P4—C2B	102.15 (16)
O2—P2—C3A	110.87 (15)	O4—P4—C3B	110.94 (15)
C9A—P2—C3A	105.85 (17)	C9B—P4—C3B	106.63 (18)
C2A—P2—C3A	107.94 (14)	C2B—P4—C3B	108.58 (15)
C2A—C1A—P1	121.3 (3)	C2B—C1B—P3	122.3 (3)
C2A—C1A—H1A	119.4	C2B—C1B—H1B	118.9
P1—C1A—H1A	119.4	P3—C1B—H1B	118.9
C1A—C2A—P2	120.2 (3)	C1B—C2B—P4	121.8 (3)
C1A—C2A—H2A	119.9	C1B—C2B—H2B	119.1
P2—C2A—H2A	119.9	P4—C2B—H2B	119.1
C4A—C3A—C8A	118.9 (3)	C4B—C3B—C8B	117.9 (3)
C4A—C3A—P2	116.6 (3)	C4B—C3B—P4	118.4 (3)
C8A—C3A—P2	124.5 (2)	C8B—C3B—P4	123.5 (3)
C3A—C4A—C5A	120.9 (4)	C3B—C4B—C5B	121.2 (4)
C3A—C4A—H4A	119.6	C3B—C4B—H4B	119.4
C5A—C4A—H4A	119.6	C5B—C4B—H4B	119.4
C6A—C5A—C4A	119.8 (4)	C6B—C5B—C4B	120.0 (4)
C6A—C5A—H5A	120.1	C6B—C5B—H5B	120.0
C4A—C5A—H5A	120.1	C4B—C5B—H5B	120.0
C5A—C6A—C7A	120.7 (4)	C7B—C6B—C5B	119.6 (3)
C5A—C6A—H6A	119.7	C7B—C6B—H6B	120.2
C7A—C6A—H6A	119.7	C5B—C6B—H6B	120.2
C6A—C7A—C8A	120.2 (4)	C6B—C7B—C8B	120.3 (4)
C6A—C7A—H7A	119.9	C6B—C7B—H7B	119.8
C8A—C7A—H7A	119.9	C8B—C7B—H7B	119.8
C3A—C8A—C7A	119.5 (4)	C3B—C8B—C7B	120.9 (4)
C3A—C8A—H8A	120.3	C3B—C8B—H8B	119.5
C7A—C8A—H8A	120.3	C7B—C8B—H8B	119.5
P2—C9A—H9A1	109.5	P4—C9B—H9B1	109.5
P2—C9A—H9A2	109.5	P4—C9B—H9B2	109.5
H9A1—C9A—H9A2	109.5	H9B1—C9B—H9B2	109.5
P2—C9A—H9A3	109.5	P4—C9B—H9B3	109.5
H9A1—C9A—H9A3	109.5	H9B1—C9B—H9B3	109.5
H9A2—C9A—H9A3	109.5	H9B2—C9B—H9B3	109.5
C11A—C10A—C15A	119.1 (3)	C15B—C10B—C11B	118.8 (3)
C11A—C10A—P1	117.6 (3)	C15B—C10B—P3	123.8 (3)
C15A—C10A—P1	123.2 (2)	C11B—C10B—P3	117.4 (3)
C12A—C11A—C10A	120.5 (4)	C12B—C11B—C10B	120.8 (4)
C12A—C11A—H11A	119.8	C12B—C11B—H11B	119.6
C10A—C11A—H11A	119.8	C10B—C11B—H11B	119.6
C13A—C12A—C11A	120.5 (4)	C13B—C12B—C11B	120.2 (4)
C13A—C12A—H12A	119.8	C13B—C12B—H12B	119.9
C11A—C12A—H12A	119.8	C11B—C12B—H12B	119.9
C12A—C13A—C14A	120.5 (4)	C12B—C13B—C14B	120.3 (4)
C12A—C13A—H13A	119.8	C12B—C13B—H13B	119.8
C14A—C13A—H13A	119.8	C14B—C13B—H13B	119.8
C13A—C14A—C15A	119.4 (4)	C13B—C14B—C15B	119.5 (4)
C13A—C14A—H14A	120.3	C13B—C14B—H14B	120.2
C15A—C14A—H14A	120.3	C15B—C14B—H14B	120.2
C10A—C15A—C14A	120.0 (4)	C10B—C15B—C14B	120.4 (4)
C10A—C15A—H15A	120.0	C10B—C15B—H15B	119.8
C14A—C15A—H15A	120.0	C14B—C15B—H15B	119.8
P1—C16A—H16A	109.5	P3—C16B—H16D	109.5
P1—C16A—H16B	109.5	P3—C16B—H16E	109.5
H16A—C16A—H16B	109.5	H16D—C16B—H16E	109.5
P1—C16A—H16C	109.5	P3—C16B—H16F	109.5
H16A—C16A—H16C	109.5	H16D—C16B—H16F	109.5
H16B—C16A—H16C	109.5	H16E—C16B—H16F	109.5
			
C3A—P2—C2A—C1A	−125.9 (7)	O1—P1—C1A—C2A	6.21 (37)
P1—C1A—C2A—P2	−167.1 (5)	O2—P2—C2A—C1A	−1.83 (36)
C16A—P1—C1A—C2A	131.7 (2)	P1—C1A—C2A—P2	−167.15 (20)
